# Drogenbezogene Probleme im psychiatrischen Konsildienst

**DOI:** 10.1007/s00115-025-01831-9

**Published:** 2025-06-18

**Authors:** Norbert Scherbaum, Udo Bonnet

**Affiliations:** 1 LVR-Universitätsklinik Essen, Klinik für Psychiatrie und Psychotherapie, Medizinische Fakultät der Universität Duisburg-Essen, Virchowstraße 174, 45147 Essen, Deutschland; 2 Klinik für Seelische Gesundheit, Ev. Krankenhaus Castrop-Rauxel, Castrop-Rauxel, Deutschland

**Keywords:** Safer use, Substitutionsbehandlung, Drogenabhängigkeit, Somatische Komplikationen, Motivierende Gesprächsführung, Safer use, Maintenance treatment, Drug dependence, Somatic complications, Motivational interviewing

## Abstract

**Hintergrund:**

Gesundheitliche Probleme beim Konsum psychoaktiver Substanzen sind ein häufiger Anlass für die Anforderung eines psychiatrischen Konsils.

**Ziel der Arbeit:**

Darstellung somatischer und psychischer Komplikationen des akuten und chronischen Konsums verschiedener Drogen (außer Alkohol und Benzodiazepinen) sowie des Vorgehens bei Konsilien.

**Material:**

Narrative Literaturübersicht.

**Ergebnisse:**

Schwerwiegende akute somatische wie auch psychische Komplikationen bei Drogenkonsum können auch bei sporadischem Konsum auftreten. Diagnostisch sind der sporadische Drogenkonsum, der schädliche Konsum und die Abhängigkeit abzugrenzen. Je nach Einzelfall sind Abstinenzempfehlung, Safer-use-Empfehlungen und motivierende Interventionen zur Aufnahme anschließender Hilfen vorzunehmen. Hierfür ist eine präzise Kenntnis der lokalen Hilfen für Drogenkonsumenten notwendig. Die medikamentöse Linderung von Entzugsbeschwerden erfolgt symptomatisch. Bei der Entzugsbehandlung sind potenzielle Komplikationen zu berücksichtigen, z. B. Entzugsdelir bei GABAergen Drogen. Bei Personen mit Opioidabhängigkeit ist eine bestehende Substitutionsbehandlung in aller Regel fortzusetzen bzw. eine solche Behandlung zu initiieren.

**Diskussion:**

Aufgaben des Konsiliars sind die kollegiale Unterstützung bei erschwerter Diagnostik sowie die Hilfe bei der Erarbeitung einer differenzierten Behandlungsempfehlung. Das suchtpsychiatrische Konsil sollte dabei nicht nur beim Management akuter klinischer Probleme Hilfe bieten, sondern für Betroffene auch die Möglichkeiten weiterer Hilfen und Behandlungen aufzeigen.

## Abstract (en)

**Background:**

The use of psychoactive substances is associated with the risk of somatic complications requiring treatment. Psychiatric consultations can be requested in this context.

**Aim:**

Description of physical and psychological complications of acute and chronic use of various drugs (except alcohol and benzodiazepines), and the procedure for consultations.

**Materials:**

Narrative literature review.

**Results:**

Severe physical and psychological complications of drug use can also occur with sporadic use. New psychoactive substances (NPS) in particular are known to have severe side effects compared to plant-based drugs with similar effects. Sporadic drug use, harmful use, and dependence must be differentiated diagnostically. Somatic complications of chronic use usually occur with dependent use, especially with intravenous drug administration. Depending on the individual case, recommendations for abstinence, safer use, or motivational interventions to take up a subsequent support program should be made. This requires precise knowledge of the local treatment network for drug users. Medication is used to alleviate withdrawal symptoms symptomatically. Potential complications must be taken into account during withdrawal treatment, e.g. withdrawal delirium and seizures in the case of GABA-ergic drugs. Maintenance treatment is the therapy of choice for opioid addicts.

**Conclusion:**

Addiction psychiatry consultations should not only offer help in the management of current clinical problems, but should also show those affected the possibilities of further care and treatment.

Der Konsum von Suchtmitteln ist mit dem Risiko psychischer und somatischer Komplikationen verbunden. Bei ausgeprägten somatischen Komplikationen kann eine Behandlung in einer körpermedizinischen Fachdisziplin notwendig sein. Dies betrifft z. B. die vital bedrohliche Atemdepression in der akuten Opioidintoxikation. Zudem sind Abhängigkeitssyndrome oft mit chronischen Folgeerkrankungen assoziiert, z. B. Hepatitis C bei intravenösem Konsum Heroinabhängiger. Akute und chronische somatische Komplikationen sind ein wesentlicher Aspekt des Risikopotenzials von Suchtmitteln [3, 12]. Die Behandlung von Menschen, die psychoaktive Substanzen konsumieren, in einer körpermedizinischen Fachdisziplin kann Anlass sein für die Anforderung eines psychiatrischen Konsils. Im Folgenden werden Problemstellungen bei Konsumenten von Suchtmitteln (außer Alkohol und Benzodiazepinen) für einen psychiatrischen Konsildienst dargestellt.

## Kontexte des psychiatrischen Konsils – akute Komplikationen des Konsums psychoaktiver Substanzen

Bei Suchtmittelintoxikationen können neben (von den Konsumenten gewünschten) psychischen Wirkungen wie Euphorie oder Entspannung schwerwiegende somatische Komplikationen auftreten mit der Notwendigkeit einer stationären Behandlung ([14]; Tab. 1). Diese Komplikationen sind z. T. Ausdruck der pharmakodynamischen Wirkung der Droge, z. B. eine Blutdruckkrise beim Konsum von Stimulanzien, z. T. Folge toxischer Beimengungen, z. B. Thrombophlebitis, Hautulzera und Muskelschäden bei intravenöser Injektion illegal produzierten und daher von Begleitsubstanzen nicht gereinigten Desomorphins („Krokodil“; [9]). Insbesondere bei Intoxikationen mit neuen psychoaktiven Substanzen (NPS) können schwerwiegende somatische Komplikationen auftreten [15, 16]. Oft sind NPS den Behandlern nicht bekannt. Zudem werden NPS in Drogenschnelltests (Enzymimmunoassay) in der Regel nicht nachgewiesen. In den USA ist der Konsum neuer synthetischer Opioide (NSO) verbreitet, insbesondere von Fentanyl(‑Analoga). Ihre im Vergleich zu Heroin oft vielfach stärkere agonistische Wirkung am µ‑Rezeptor des Endorphinsystems führt zu einer erhöhten Drogenmortalität [15]. Akute Komplikationen bei Intoxikationen können auch bei sporadischem Konsum auftreten, z. B. beim Konsum von Ecstasy und Stimulanzien als „Club Drugs“ oder beim sog. „ChemSex“ von Männern, die Sex mit Männern (MSM) haben [5].

**Tab. 1:** Somatische Komplikationen bei Drogenkonsum

Substanz	Akute Komplikationen (Intoxikation)	Komplikationen bei chronischem Konsum
Amphetamine	Herzrhythmusstörungen, Herzinfarkt, Schlaganfall, zerebrale Krampfanfälle	Gewichtsabnahme, Karies und Zahnbruch, Hautinfektionen, Risiken des intravenösen Konsums^a^
Cannabis	Delir, Herzrhythmusstörungen, Herzinfarkt, Schlaganfall	Bei inhalativem Konsum: chronische Bronchitis, karzinogen; Cannabis-Hyperemesis-Syndrom
Synthetische Cannabinoide (z. B. „Spice“)	Delir, epileptische Anfälle, Dyspnoe, Rhabdomyolyse; Koma mit Atemdepression, Hepatopathie, Nephropathie, Elektrolytstörungen, Hyperthermie, kardiovaskuläre Ereignisse, Enzephalopathien	Mutmaßlich analog zu Cannabis
Cathinone (z. B. Mephedron)	Delir, epileptische Anfälle, Rhabdomyolyse, Koma mit Atemdepression, Hepatopathie, Nephropathie, Elektrolytstörungen, Hyperthermie, kardiovaskuläre Ereignisse, Enzephalopathie	Anämie, Panzytopenie
„Ecstasy“ (MDMA)	Kiefersperre, Hyperthermie, Rhabdomyolyse, disseminierte intravasale Gerinnung, Nierenversagen, Hirnödem, akutes Leberversagen, Herzrhythmusstörungen, epileptische Krampfanfälle, Hirnblutung bei Blutdruckkrise	Daten zu somatischen Komplikationen betreffen vorrangig akute Komplikationen
Fliegenpilz	Delir, Übelkeit, Erbrechen, Sprachstörungen, Herzrasen, Muskelzuckungen, Atemlähmung	Meist sporadischer Konsum
GHB (γ-Hydroxybuttersäure)	Übelkeit, Erbrechen, Sedierung bis zur Bewusstlosigkeit, Koma mit Atemdepression	Daten zu somatischen Komplikationen betreffen vorrangig akute Komplikationen
Heroin, Opioidanalgetika, neue synthetische Opioide	Erbrechen, Atemlähmung, Herzrhythmusstörungen, Koma mit Atemdepression, Enzephalopathien	^a^Risiken des intravenösen Konsums
Ketamin	Erbrechen, Bauchschmerzen („K-cramps“), Bewusstlosigkeit, in Kombination mit Opiaten Koma mit Atemdepression, Blutdruckkrise (bei Mischkonsum mit Stimulanzien)	Ulzerative Zystitis
Kokain	Delir, epileptische Anfälle, Hyperthermie, Herzinfarkt, Hirnblutung bei Blutdruckkrise, Atemdepression; Herzmuskelschädigung, bei inhalativem Kokain: Chorea („Crack Dance“)	Gewichtsabnahme, verschlechterter Zahnstatus, Läsionen der Nasenscheidewand (intranasale Applikation); ^a^Risiken des intravenösen Konsums
Lachgas	Übelkeit, Gleichgewichtsstörungen, Bewusstlosigkeit, Atemstillstand; Verätzungen im Mundbereich bei Inhalation aus Lachgasbehälter	Myeloneuropathie mit Sensibilitätsstörungen und Lähmungen, perniziöse Anämie
Pflanzliche Halluzinogene	Erheblicher Brechreiz (Meskalin), Übelkeit (Psilcybin), Serotoninsyndrom (bei Monoaminoxidase[MAO]-Hemmung)	Meist sporadischer Konsum
Schnüffelstoffe (unterschiedlich bei verschiedenen Schnüffelstoffen)	Übelkeit, Reizung der Atemwege, Bewusstlosigkeit, Ersticken (bei Inhalation aus Tüten), Atemstillstand, toxische Wirkung auf Herz, Lunge („Benzinpneumonie“), Leber (hepatisches Koma), Nieren	Anämie, Panzytopenie, Benzol-Leukämie, periphere Polyneuropathie, karzinogen, organische Persönlichkeitswandlung, Lähmungen, kognitiver Abbau, Verschlechterung arterieller Hypertonie und Diabetes mellitus

^a^Risiken des intravenösen Konsums: Infektion mit Hepatitis B und C sowie mit dem HIV („human immunodeficiency virus“), Endokarditis, Spritzenabszess

– Allfällige Intoxikationssymptome wie Störungen der motorischen Koordination, Übelkeit, Schwindel, Stürze, verminderte Fahrsicherheit etc. werden nicht aufgeführt

– Bei Intoxikation mit Stimulanzien (Amphetaminen, Cathinonen, Ecstasy, Kokain) Gefahr des Serotoninsyndroms (vor allem bei Mischkonsum), bei chronischem Konsum Verschlechterung von arterieller Hypertonie und Diabetes mellitus

**Tab. 2:** Klinisches Management bei Intoxikation und Entzugssyndrom bei verschiedenen psychoaktiven Substanzen [8]

Psychische Störungen durch	Strategien bei Intoxikationen	Strategien bei Entzugssyndromen
Opioide (F11.x)	Überwachung der Vitalwerte, Naloxon i.v.; ggf. Intubation und Beatmung	*Heroinentzug:* Umstellung auf langwirksames Opioid, z. B. Methadon; dann sukzessive Abdosierung; symptomorientierte Begleitmedikation, z. B. nichtsteroidale Antirheumatika (Schmerzen), Antidepressiva mit sedierender Komponente (innere Unruhe, Schlafstörung), Clonidin (sympathische Rebound-Hyperaktivität)
Cannabinoide (F12.x)	Überwachung der Vitalwerte, in der Regel unkompliziert, bei komplizierten Intoxikationen mit Panikattacken oder Erregungszuständen Benzodiazepine; die intoxikationsbedingte Cannabispsychose oder -manie remittiert in der Regel auch ohne Neuroleptika im Verlauf, ggf. atypische Neuroleptika	Nichtmedikamentöses Symptommanagement, z. B. körperliche Bewegung; bei ausgeprägten Entzugssymptomen mit innerer Unruhe, Reizbarkeit und Schlafstörung sedierende Neuroleptika wie Promethazin oder bei Nichtansprechen stationär auch vorübergehend Gabapentinoide oder Benzodiazepine
Kokain (F14.x)	Überwachung der Vitalwerte, in der Regel unkomplizierte Ausnüchterung, bei Intoxikationspsychosen Benzodiazepine; bei Herzrhythmusstörungen kardiologische Abklärung, bei epileptischen Anfällen oder extrapyramidalen Symptomen neurologische Abklärung	Nichtmedikamentöses Symptommanagement; bei dominierenden quälenden Symptomen wie Unruhe oder Schlaflosigkeit sedierende Neuroleptika; typisch ist ein etwa 5 bis 7 Tage anhaltender adynamer Zustand („cocaine crash“). CAVE: Suizidalität (Entzugsbehandlung im Schutzraum einer Klinik)
Stimulanzien (F15.x)	Überwachung der Vitalwerte, in der Regel unkomplizierte Ausnüchterung, bei Erregungszuständen oder Intoxikationspsychose Benzodiazepine und/oder atypische Neuroleptika; bei Herzrhythmusstörungen kardiologische Abklärung, bei epileptischen Anfällen oder extrapyramidalen Symptomen neurologische Abklärung	Nichtmedikamentöses Symptommanagement; zur medikamentösen Linderung von Entzugsbeschwerden trizyklische Antidepressiva oder Bupropion, nachrangig auch Benzodiazepine
Halluzinogene (F16.x)	Überwachung der Vitalwerte, in der Regel unkomplizierte Ausnüchterung, bei Erregungszuständen oder Intoxikationspsychose am ehesten Benzodiazepine (Neuroleptika gelten als unwirksam oder sogar symptomverschlimmernd)	Ein Entzugssyndrom ist nicht zu erwarten

Neben der diagnostischen Zuordnung der somatischen Symptome zu bestimmten Substanzen dürfte die Frage nach einer substanzbedingten Störung und deren etwaige Behandlung Gegenstand der Konsilanfrage sein. Dass die diagnostischen Kriterien nach ICD-10 (International Statistical Classification of Diseases and Related Health Problems 10) weitgehend auf Eigenangaben der Betroffenen beruhen, kann die Diagnostik eines Abhängigkeitssyndroms erschweren. Eine vertrauensvolle Atmosphäre mit Zusicherung der ärztlichen Schweigepflicht (abgesehen von den konsilanfordernden Ärzten) ist daher wichtig. Bei illegalen Suchtmitteln ist darauf hinzuweisen, dass die ärztliche Schweigepflicht auch gegenüber Strafverfolgungsbehörden gilt. Bei begrenzter Kooperation der Betroffenen kann gegebenenfalls nur ein schädlicher Konsum diagnostiziert werden, auch wenn der Verdacht auf ein Abhängigkeitssyndrom besteht.

(Abhängige) Suchtmittelkonsumenten sind erhöht suizidgefährdet [18]. Dies kann unmittelbar durch die Substanzwirkung bei mangelnder Kontrolle suizidaler Impulse bedingt sein oder durch eine aversive Affektlage mit Anhedonie oder niedergedrückter Stimmung im Entzug. Insbesondere im Kokainentzug ist an die erhöhte Suizidgefahr zu denken (Kokain-„Crash“). Zudem können Affekte der Verzweiflung und Hoffnungslosigkeit durch eine desolate gesundheitliche und soziale Situation bedingt sein. Daher ist die Evaluation der Suizidalität obligater Teil des psychiatrischen Konsils – auch wenn diese nicht explizit im Konsilauftrag erbeten wurde.

Das therapeutische Vorgehen richtet sich nach der diagnostischen Einschätzung (Tab. 2). Insbesondere bei sporadischem Konsum sollten neben der grundsätzlichen Empfehlung, dass nur die Abstinenz risikofrei ist, auch Strategien zum „safer use“ vermittelt werden. Solche Empfehlungen beruhen auf der Kenntnis der spezifischen Risiken der jeweiligen Substanzen. Zudem werden die individuellen Risiken der Betroffenen, z. B. Vorerkrankungen, familiäre Belastungen für bestimmte Erkrankungen, berücksichtigt. Für Konsumenten von Cannabis wurden detaillierte „safer use guidelines“ entwickelt [7, deutschsprachige Version 4]. Die 12 Empfehlungen betreffen z. B. die Vermeidung des gleichzeitigen Konsums mehrerer Suchtmittel, kein Führen eines Fahrzeuges bei Intoxikation sowie die Wahl der weniger schädlichen Applikationsform (hier orale Einnahme vor inhalativer Einnahme). Analoge Empfehlungen sind auch bei anderen Suchtmitteln sinnvoll. Bei intravenös applizierbaren Drogen wie Heroin wird die Vermeidung der intravenösen Einnahme, insbesondere unter hygienisch bedenklichen Umständen, empfohlen. Ziel eines solchen Gesprächs ist auch die Prävention der Entwicklung einer Abhängigkeit.

Bei substanzbedingten Störungen ist eine Behandlung indiziert – gegebenenfalls nach dem Abklingen der akuten Komplikationen. Sofern keine oder eine schwach ausgeprägte Behandlungsmotivation besteht, ist ein Gespräch nach den Prinzipien des „motivational interviewing“ sinnvoll [11]. Hierbei können im Kontext eines Konsils die aktuellen Komplikationen ein Anknüpfungspunkt sein. Bei Komplikationen besteht eine kognitive Dissonanz zwischen dem Wunsch nach der Drogenwirkung und dem der Gesunderhaltung. Sofern Patienten zur Aufnahme von Hilfen motiviert sind, sollen diesen auch konkret Ansprechpartner genannt werden, z. B. von Drogenberatungsstellen, Substitutionsambulanzen, Klinikambulanz und Entzugsstation(en). So wird die tatsächliche Inanspruchnahme von Hilfen wahrscheinlicher. Konsiliarisch tätige Ärzte müssen sich also in lokalen Hilfesystem für Drogenkonsumenten auskennen. Gegebenenfalls ist auch die Übernahme in eine psychiatrische Klinik nach Abklingen der somatischen Behandlungsbedürftigkeit indiziert.

## Kontexte des psychiatrischen Konsils – chronische Komplikationen des Konsums psychoaktiver Substanzen

Einige Suchtmittel haben eine toxische Wirkung auf den Organismus, dessen Folgen insbesondere bei chronischem Gebrauch auftreten. Hierzu zählt z. B. die Schädigung langer Nervenbahnen bei der chronischen Einnahme von Lachgas mit Sensibilitätsstörungen und Lähmungen [14]. Somatische Folgeerkrankungen beim Rauchen von Cannabis („Joint“) wie z. B. chronische obstruktive Lungenerkrankung (COPD) sind (analog zum Zigarettenrauchen) durch das Entstehen von Beimengungen bei Verbrennungsprozessen zu erklären [2]. Andere Suchtmittel sind zwar nicht per se toxisch. Ihre Einnahme erfolgt aber unter hygienisch problematischen Bedingungen, die wiederum das Risiko für somatische Erkrankungen erhöht. Dies gilt insbesondere für die Folgen des intravenösen Drogenkonsums wie Spritzenabszess, Endokarditis oder Hepatitis C (Tab. 1). Schließlich sind Menschen mit Drogenabhängigkeit in ihren Lebensmilieus auch Opfer gewalttätiger Übergriffe mit gesundheitlichen Folgen wie Knochenbrüchen.

Chronische Komplikationen des Konsums psychoaktiver Substanzen sind meist mit einem abhängigen Suchtmittelkonsum verbunden, sodass die Behandlung des Entzugssyndroms einschließlich der Einleitung einer Anschlussbehandlung zu klären sind.

### Entzugsbehandlung

Ziele der Entzugsbehandlung sind insbesondere die medikamentöse Linderung der Entzugsbeschwerden (Tab. 2), die Diagnostik etwaiger komorbider psychischer Störungen sowie motivationale Interventionen zur Aufnahme einer Anschlussbehandlung (siehe „motivational interviewing“). Nur für den Opioidentzug besteht eine hinreichende Evidenz zur spezifischen Entzugsbehandlung (α_2_-Agonisten; Opioidsubstitute wie Methadon oder Buprenorphin in absteigender Dosis; [8]). Bei anderen Abhängigkeiten werden symptomatisch Medikamente eingesetzt, z. B. Neuroleptika oder sedierende Antidepressiva bei Schlafstörung [8]. Bei Opioidabhängigkeit ist allerdings kritisch zu prüfen, ob in dieser Situation eine Entzugsbehandlung überhaupt indiziert ist und nicht vielmehr eine Substitutionsbehandlung, die auch eine suffiziente Behandlung der somatischen Erkrankung erleichtert. Ebenfalls wird bei Schwangeren mit Opioidabhängigkeit eine Substitutionsbehandlung in den Richtlinien der Bundesärztekammer (BÄK) empfohlen [1].

Zu den jeweiligen Entzugssyndromen bei verschiedenen Drogen sowie deren medikamentöse Linderung sei auf entsprechende Übersichtsarbeiten verwiesen [8]. Auf folgende Komplikationen beim Management von Entzugssyndromen sei jedoch hingewiesen:

- Auftreten von Delir und Entzugskrampfanfällen beim Entzug GABAerger Substanzen (Alkohol, Benzodiazepinen, Z‑Drugs, Gabapentinoide, GHB; Propofol, Chloralhydrat etc.),
- Übersehen akuter psychiatrischer Erkrankungen bei gegebenenfalls raptusartig hervorbrechenden Erregungszuständen in Intoxikation oder Entzug,
- Auftreten von Rhabdomyolyse und Elektrolytstörungen bei allen schweren Intoxikationen sowie deren weitere Verschlechterung bzw. Demaskierung im Entzug,
- Auftreten von Enzephalopathien (selten) – meistens durch Kontaminationen bei intravenösem Konsum, z. B. von Heroin oder Kokain,
- komorbide Hirnblutungen durch vorhergehenden Sturz oder Schlägerei,
- komorbide periphere Lähmungen, z. B. Radialisparese („Parkbanklähmung“) beim langen schlafenden Verharren bei ungünstiger Lagerung,
- schwer voraussehbare Entzugsverläufe bei polyvalentem (abhängigen) Konsum.

### Mittelfristige (subakute) Behandlung

Auf die Entzugsbehandlung folgt bei den meisten substanzbezogenen Abhängigkeiten die rehabilitative Behandlung in Suchtfachkliniken, z. T. auch als ambulante rehabilitative Behandlung. Nur bei der Opioidabhängigkeit gibt es die Möglichkeit der Substitutionsbehandlung mit Opioidagonisten wie Methadon oder Buprenorphin. Zum Teil dürften sich die entsprechenden Patienten bereits in einer Substitutionsbehandlung befinden. Grundsätzlich ist eine vorbestehende Medikation, hier die Substitutionsbehandlung, in stationärer Behandlung fortzusetzen, sofern nicht aktuell eine Kontraindikation aufgetreten ist. Ohnehin würde das abrupte Absetzen eines Substitutionsmittels ein Entzugssyndrom auslösen, das die Therapie der komorbiden somatischen Erkrankung erschwert. In somatischen Kliniken sollten daher insbesondere Methadon-Razemat, Levo-Methadon und Buprenorphin verfügbar sein. Hierbei sind Methadon und Buprenorphin nicht abrupt austauschbar. Dosierungen von Levo-Methadon und Methadon-Razemat verhalten sich wie 1:2 (z. B. 40 mg Levomethadon entspricht 80 mg Methadon-Razemat). Im Übrigen ist wegen der unterschiedlichen Konzentration verschiedener Methadonlösungen die Dosisangabe in mg der Dosisangabe in ml vorzuziehen, um die Risiken für Patienten durch fehlerhafte Dosierungen zu minimieren. Sofern Patienten in Substitutionsbehandlung über keinen Substitutionsausweis verfügen, ist eine Kontaktaufnahme mit der substituierenden Praxis sinnvoll. Sofern dies nicht gelingt, sollte eine fraktionierte Gabe des Substitutionsmedikamentes erfolgen, z. B. beginnend mit 20 mg Methadon-Razemat und weiteren Gaben am gleichen Tag bei objektiven Entzugssymptomen wie erweiterte Pupillen, Tachykardie, Erhöhung des sympathischen Blutdrucks oder verstärktem Schwitzen. Die Gesamtdosis des ersten Tages ist dann die Startdosis des zweiten Tages. Diese Dosis wird dann orientiert an Entzugsbeschwerden in den Folgetagen schrittweise erhöht. Als Bezugspunkte der Eindosierung möge die Angabe dienen, dass 40 mg Methadon-Razemat für opioidnaive Personen als lebensbedrohlich gelten. Bei der Gabe von Opioiden bei gleichzeitiger Gabe anderer Medikamente ist insbesondere auf die Gefahr der Verlängerung der QTc-Zeit zu achten als Indikator für das Risiko potenziell lebensbedrohlicher Herzrhythmusstörungen sowie auf die gegenseitige Verstärkung sedierender Wirkungen von Medikamenten.

## Der psychiatrische Konsildienst im Behandlungsnetzwerk

Nach dem epidemiologischen Suchtsurvey konsumierten 9,6 % Personen der erwachsenen Bevölkerung in Deutschland in den letzten 12 Monaten mindestens eine Droge (einschließlich Cannabis; [13]). Es liegen den Autoren allerdings keine Zahlen vor, wie regelhaft für Personen mit Drogenkonsum psychiatrische Konsile angefordert werden. Analog zur Wirkung des ärztlichen Ratschlags, angesichts von Folgeproblemen mit dem Rauchen von Zigaretten aufzuhören [17], ist die Annahme eines „teachable moment“ auch bei Komplikationen bei Drogenkonsum naheliegend. Das Spektrum der Ziele reichen hierbei je nach motivationalem Zustand der Patienten von der Risikoaufklärung bzw. Empfehlungen zum „safer use“, zur (Sekundär‑)Prävention gegen die Entwicklung einer Abhängigkeit, bis hin zur Einleitung einer substanzspezifischen Behandlung bei manifestem Abhängigkeitssyndrom. Auf diese Weise können psychiatrische Konsile bei Personen mit Drogenkonsum helfen, die Unterbehandlung von Menschen mit substanzbedingten Störungen („treatment gap“; [10]) zu vermindern.

Angesichts der aktuellen Probleme bei der Finanzierung der kommunalen Suchthilfe [6] sowie der Sicherstellung der Substitutionsbehandlung an manchen Orten besteht allerdings die Gefahr, dass zur Verhaltensänderung motivierte Patienten leider keine fachspezifische Unterstützung zeitnah und wohnortnah erhalten.

## Fazit für die Praxis

- Risiken von Suchtmitteln betreffen psychische und somatische Komplikationen.
- Komplikationen des Suchtmittelkonsums sind ein Anknüpfungspunkt für ein motivationales Gespräch.
- Eine Empfehlung zum „safer use“ ist sinnvoll.
- Für konkrete Empfehlungen zu weiteren Hilfen ist eine präzise Kenntnis der lokalen Hilfen für Personen mit Drogenkonsum notwendig.

## References

[1] BÄK (2023) Richtlinie Richtlinie der Bundesärztekammer zur Durchführung der substitutionsgestützten Behandlung Opioidabhängiger. https://www.bundesaerztekammer.de/fileadmin/user_upload/BAEK/Themen/Public_Health/Richtlinien/Richtlinie-BAEK-Substitution_16.02.2023.pdf. Zugegriffen: 6. Jan. 2025 10.1055/a-1173-9588 32610357

[2] Bonnet U, Specka M, Scherbaum N (2016) Häufiger Konsum von nicht-medizinischem Cannabis – Gesundheitliche Folgen und Wirkung der Entzugsbehandlung. Dtsch Med Wochenschr 141:126–131 26800074 10.1055/s-0041-106313

[3] Bonnet U, Specka M, Soyka M, Alberti T, Bender S, Hilger J, Hillemacher T, Kuhlmann T, Kuhn J, Lüdecke C, Reimer J, Schneider U, Schroeder W, Stuppe M, Wiesbeck G, Wodarz N, Scherbaum N (2022) Abwägung von Nutzen und Schädlichkeit von berauschenden und schmerzlindernden Substanzen aus der Perspektive von deutschen Suchtmedizinern. Fortschr Neurol Psychiatr 90:19–29 33634461 10.1055/a-1363-0223

[4] Deutsche Hauptstelle für Suchtfragen (DHS) (Hrsg.) (2024): Care Instructions. Für einen bewussten Umgang mit Cannabis. Hamm. https://www.dhs.de/fileadmin/user_upload/BZGA-24-05865__Faltblatt_Cannabis_%E2%80%93_Care_Instructions_BFREI.pdf. Zugegriffen: 6. Jan. 2025

[5] Deimel D, Bohn A, Sander D, Scherbaum N, Schecke H (2024) Substanzkonsum im sexuellen Kontext („Chemsex“) bei Männern, die Sex mit Männern haben – Ergebnisse des „German Chemsex Survey“. Suchttherapie 25:83–91

[6] Deutsche Hauptstelle für Suchtfragen (2024) Finanzierung der Suchtberatungsstellen in Deutschland. Ergebnisse einer bundesweiten Umfrage der Deutschen Hauptstelle für Suchtfragen e. V. (DHS). Hamm. https://www.dhs.de/fileadmin/user_upload/2024-09-26-Bericht_zur_Finanzierung_der_Suchtberatung_FINAL.pdf. Zugegriffen: 7. Jan. 2025

[7] Fischer B, Robinson T, Bullen C, Curran V, Jutras-Aswad D, Medina-Mora ME, Pacula RL, Rehm J, Room R, Brink WVD, Hall W (2022) Lower-Risk Cannabis Use Guidelines (LRCUG) for reducing health harms from non-medical cannabis use: A comprehensive evidence and recommendations update. Int J Drug Policy 99:103381 34465496 10.1016/j.drugpo.2021.103381

[8] Gouzoulis-Mayfrank E, Scherbaum N (2025) Drogenabhängigkeit. In: Voderholzer U, Hohagen F (Hrsg) Therapie psychischer Erkrankungen – State of the Art, 20. Aufl. Urban & Fischer, München Jena, S 61–79

[9] Katselou M, Papoutis I, Nikolaou P, Spiliopoulou C, Athanasetis S (2014) A „Krokodil“ emerges from the murky waters of addiction. Abuse trends of an old drug. Life Sci 102:81–87 24650492 10.1016/j.lfs.2014.03.008

[10] Mack S, Jacobi F, Gerschler A, Strehle J, Höfler M, Busch MA, Maske UE, Hapke U, Seiffert I, Gaebel W, Zielasek J, Maier W, Wittchen H‑U (2014) Self-reported untilization of mental health services in the adult German population—evidence for unmet needs? Results of the DEGS-1-Mental Health Module (DEGS1-MH). Int J Methods Psychiatr Res 23:289–303 24687693 10.1002/mpr.1438 PMC6878535

[11] Miller WR, Rollnick S (2015) Motivierende Gesprächsführung, 3. Aufl. Lambertus, Freiburg

[12] Nutt DJ, King LA, Phillips LD, Independent Scientific Committee on Drugs (2010) Drug harms in the UK: a multicriteria decision analysis. Lancet 376(9752):1558–1565 21036393 10.1016/S0140-6736(10)61462-6

[13] Rauschert C, Möckl J, Seitz N‑N, Wilms N, Olderbak S, Kraus L (2022) Konsum psychoaktiver Substanzen in Deutschland – Ergebnisse des Epidemiologischen Suchtsurvey 2021. Dtsch Ärztebl Int 2022(119):527–534 10.3238/arztebl.m2022.0244 PMC9677535 35791270

[14] Scherbaum N (2024) Das Drogentaschenbuch, 7. Aufl. Thieme, Stuttgart

[15] Scherbaum N, Bonnet U (2024) Die Herausforderungen für die psychiatrische Versorgung durch synthetische Drogen. Nervenarzt 95:818–823 39186107 10.1007/s00115-024-01705-6

[16] Schifano F, Vento A, Scherbaum N, Guirgis A (2023) Stimulant and hallucinogenic novel psychoactive substances; a review. Expert Rev Clin Pharmacol. 10.1080/1751122433.2023.2279192 37968919 10.1080/17512433.2023.2279192

[17] Stead LF, Buitrago D, Preciado N, Sanchez G, Hartmann-Boyce J, Lancaster (2013) Physician advice for smoking cessation (Review). Cochrane Database Syst Rev 5:CD165 10.1002/14651858.CD000165.pub4 PMC7064045 23728631

[18] Stephenson M, Edwards AC (2024) Investigating Associations of Substance Use and Dependence With Planned Versus Unplanned Suicide Attempt. J Stud Alcohol Drugs 85:339–348 38227385 10.15288/jsad.23-00205 PMC11095497

